# Schistosomiasis Japonica Control in Domestic Animals: Progress and Experiences in China

**DOI:** 10.3389/fmicb.2017.02464

**Published:** 2017-12-14

**Authors:** Zhiguo Cao, Yinyin Huang, Tianping Wang

**Affiliations:** ^1^Department of Immunology and Pathogenic Biology, Health Science Center, Xi’an Jiaotong University, Xi’an, China; ^2^Anhui Provincial Institute of Schistosomiasis Control, Hefei, China

**Keywords:** schistosomiasis japonica, domestic animal, control, process, experience

## Abstract

Schistosomiasis japonica, caused by *Schistosoma japonicum*, is an endemic, zoonotic parasitic disease. Domestic animals, particularly bovines, are thought to play an important role in transmission of the disease. Historically, China was the country mostly severely impacted by schistosomiasis japonica, but now prevalence and morbidity have been greatly reduced. Since the mid-1950s when China launched the National Schistosomiasis Control Program, the control of schistosomiasis in domestic animals has been carried out almost synchronously with that of human schistosomiasis, and this concept has been proven to be effective. Generally, the campaign of schistosomiasis japonica control in domestic animals in China went through four phases over the past six decades, namely, the large-scale epidemiological investigation phase, the case screening and small-scale chemotherapy phase, the mass chemotherapy phase, and the infection source control phase. These distinct phases were responsive to changing disease epidemiology, socioeconomic development, and technological advances, resulting in successful attainment of disease control goals.

## Introduction

Schistosomiasis japonica, an endemic, zoonotic parasitic disease caused by *Schistosoma japonicum* (*S. japonicum*), is found in China, the Philippines, Indonesia and Japan, with China historically being the most heavily endemic of the four countries (Collins et al., 2012; Chen, 2014). Large-scale epidemiological surveys carried out after foundation of the People’s Republic of China in 1949 estimated 14,300 km^2^ within the mainland were infested with *Oncomelania hupensis* (*O. hupensis*), the intermediate host of *S. japonicum.* More than 11.60 million people and 1.2 million bovines (cattle and water buffaloes) were estimated to be infected with the parasite (Minggang and Feng, 1999).

Unlike other species of schistosomes, over 40 species of domestic and wild animals, including bovines, pigs, sheep and dogs, can act as reservoir hosts of *S. japonicum*, which increases the difficulty of infection control (MOH, 2000; Lin et al., 2011). Schistosomiasis japonica can cause serious losses of animal husbandry production. Additionally, the infected domestic animals, especially bovines, can function as sources of schistosomiasis japonica infection, responsible for up to 75% of disease transmission (Guo et al., 2001; Gray et al., 2009). Therefore, the ultimate goal to eliminate schistosomiasis japonica across China would be impossible without effectively controlling *S. japonicum* infections in domestic animals. In the past 60 years, China has promoted synchronous control strategies that were adaptive to the socioeconomic and technological advances as well as epidemiological characteristics for *S. japonicum* infection in both humans and domestic animals. These distinctive efforts have yielded great progress: the overall prevalence of schistosomiasis japonica is less than 1% in humans and livestock populations, and all endemic provinces have reached the criteria of transmission control (Cao et al., 2011, 2016; Zhang L.J. et al., 2016).

## The Phases of Schistosomiasis Japonica Control in Domestic Animals in China

### Large-Scale Epidemiological Investigation Phase (Mid- to Late 1950s)

This was the initial phase of schistosomiasis japonica control in domestic animals in China, when the main tasks were (1) to understand the endemic status in livestock through epidemiological surveys and (2) to preserve the health of bovines through timely examination and treatment so that these animals could be used in agriculture. The prevalence situation was generally characterized by the middle to late 1950s, and the statistics of 1958 reported that some 1.2 million bovines were infected with schistosomiasis japonica, and another 5 million were at high risks of infection, with infection prevalence ranging from 5.3 to 85.9% in bovines in this period. Pigs, sheep, and dogs living in the endemic regions were also seriously affected by schistosomiasis japonica, and the mean infection prevalence of these domestic animals reached up to 50% in certain endemic areas (Mao, 1990; Shen, 1992).

### Case Screening and Small-Scale Chemotherapy Phase (1960s to Early 1980s)

Case screening and chemotherapy on small scale were emphasized during this phase in China, and simple schistosomiasis japonica control in domestic animals was extended from bovines to all susceptible livestock. The goal of this practice was effective control of the infection sources through controlling environmental contamination from the excreted schistosome eggs in the feces of infected animals, while ensuring the health of livestock was secondary. Chemical drugs used to treat domestic animal schistosomiasis included antimonyl potassium tartrate, furpromide, hexachloroparaxylene, and metrifonate. However, these drugs were found to have severe lethal side effects, long term treatment and poor efficacy that had to be weighed against the benefits for the infected animals (Mao, 1990).

### Mass Chemotherapy Phase (Mid-1980s to 2003)

Since the mid-1980s, the mass chemotherapy strategy was adopted in China. At this time, praziquantel, the anti-schistosomiasis drug, was synthesized, and the drug possessed the characteristics of low toxicity, high efficacy, short treatment course and ease of administration. This strategy had been effectively implemented under the support of the World Bank Loan Project for Schistosomiasis Control in China (1992–2001). Mass use of praziquantel contributed greatly to reduction of both the prevalence and intensity of schistosome infections (Chen, 2005). By the end of the program, the number of infected bovines were decreased by 97.5% from 1.2 million in the 1950s to 30,000 in 2001 (Chen et al., 2002). *S. japonicum* infections in bovines, pigs, sheep, dogs, and other domestic animals in endemic provinces were reduced to 9.09, 2.09, 2.46, 1.87, and 3.33% by the second nationwide schistosomiasis survey carried out in 1995 from 13.29, 3.11, 10.4, 2.62, and 5.64% as measured in the first nationwide schistosomiasis survey in 1989, respectively (MOH, 1993, 1998). According to the third nationwide schistosomiasis survey conducted in 2004, prevalence of *S. japonicum* in the aforementioned domestic animals was further reduced on average to 4.37% in bovines, 0.27% in pigs, 1.44% in sheep, and no positives were detected in the 539 sampled domestic animals including dogs, horses, and donkeys (MOH, 2006). Although mass drug administration had greatly reduced the prevalence and morbidity of schistosomiasis, it was unable to prevent reinfection, resulting in difficulty in maintaining and consolidating the previous efforts and achievements (Liu et al., 1999; Zhou et al., 1999; Ross et al., 2002; Li and Lin, 2007).

### Infection Source Control Phase (2004 to Present)

In order to solve the problem of reinfection in domestic animals, China decided to shift from the previous strategy of mass chemotherapy to integrated control of the infection sources after 2004. The underlying rationale of this strategy was to limit the contamination of the environment with schistosome eggs by reducing the roles of domestic animals, especially bovines, as sources of infection for *O. hupensis*. The contamination-prevention measures included: (1) replacing bovines with tractors for farming; (2) fencing of pastures to prevent bovines from grazing on grasslands with *O. hupensis*; (3) raising domestic animals in pens; (4) building biogas digesters to handle excreta of domestic animals. These comprehensive actions proved greatly effective, and led to significant decrease of *S. japonicum* infection in domestic animals. These reductions were achieved in a relatively short time period and resulted in infections being maintained at lower level (Wang et al., 2009a; Cao et al., 2012). By 2012, the prevalence of *S. japonicum* in domestic animals in endemic areas across the nation was reduced to 0.58% in bovines, 0.57% in sheep, and 0.01% in other domestic animals (pigs, dogs, horses, etc…) (Li H. et al., 2014). The prevalence of bovine schistosomiasis was further reduced to 0.06% by 2015, when only 315 infected bovines were reported throughout China. Domestic animal infections were decreased by 98.7% compared to that in 2003 when 24461 bovines were infected. These results demonstrate that the overall prevalence of schistosomiasis japonica in domestic animals were reduced to the lowest threshold observed in China (Zhang L.J. et al., 2016).

## Key Experiences Concerning Control of Schistosomiasis Japonica in Domestic Animals in China

### Adhering to the Principle of Synchronous Control of Schistosomiasis Japonica in Humans and Domestic Animals

Many species of domestic animals can play important roles in transmission of the disease, and humans and domestic animals may be concurrently involved. Therefore, failure control of the transmission either in humans or in domestic animals will make it hard to reduce the overall endemic situation of schistosomiasis japonica within certain regions. Therefore, the Chinese government has pursued synchronous control of schistosomiasis japonica in humans and domestic animals since initiation of the National Schistosomiasis Control Program in the mid-1950s, and this concept has been proven to be effective over the past 60 years of control practice (Wang et al., 2008).

### Adhering to the Scientific Control Strategies Which Kept Pace with Time

Transmission of schistosomiasis japonica is generally affected by biological, environmental, and social factors. China’s efforts to reduce schistosomiasis japonica prevalence over more than half a century proves that effective and successful control of the disease is strongly attributable to the strategies planned in scientific manner. The remarkable achievements in China in control of domestic animal schistosomiasis have been the focus of worldwide attention, this should be responsible for the scientific control strategies that were distinctively taken in different periods, and these strategies are in line with the change of socio-economic settings, scientific and technological levels as well as endemic status of schistosomiasis japonica. The current achievements in schistosomiasis japonica control in domestic animals would be impossible without the scientific control strategies in China (Yang and Yang, 1996; Wang et al., 2009a,b,c).

### Targeting Key Infection Sources

Transmission of schistosomiasis japonica is attributable to a variety of domestic animal infection sources, yet different animal species may contribute differently to disease transmission. Identifying the main source of infections allows control programs to prioritize these key infection sources as control resources are limited. Studies in the early period of schistosomiasis control in China indicated that bovines are the most important reservoirs of disease transmission, therefore control of bovine schistosomiasis has been a top priority in the Chinese National Schistosomiasis Control Program (Mao, 1990; Jiang and Guo, 2009; Li Y.S. et al., 2014). Many years of efforts targeting at the control of bovine infection demonstrated that the overall prevalence of schistosomiasis japonica was remarkably decreased in a short period following effective control of bovine schistosomiasis in endemic areas across the nation (Lei et al., 2015; Zhang S.Q. et al., 2016).

### Adapting Control Measures to Local Conditions

Schistosomiasis japonica is widely endemic in the south of China, and environment characteristics, endemic features, infection sources and socioeconomic levels vary considerably among endemic areas. Accordingly, China insists on schistosomiasis control measures that are tailored to local conditions. For example, the current control strategy is targeted at management of infection sources in domestic animals, and the major measures include replacing bovines with farm machines, management of domestic animals by fencing of pastures or raising them in pens, and so forth. Nevertheless, these measures are not necessarily appropriate for all endemic regions, hence, they are optional, allowing customization of control strategies to local conditions and the endemic nature of schistosomiasis japonica in each areas (Zhou et al., 2011).

### Executing Schistosmiasis Control by Laws and Regulations

The Chinese government attaches great importance to the control of schistosomiasis japonica, and issued a series of laws and regulations, including the Law of the People’s Republic of China on the Prevention and Treatment of Infectious Diseases, and Regulations on Prevention and Control of Schistosomiasis, in order to effectively facilitate preventive and control measures. In addition, local government at all levels within endemic areas also released a series of regulations and policies to ensure responsibilities of administrators involved in the control affairs and legal implementation of the measures proved to be effective in schistosomiasis japonica control (Wang, 2006).

### Adhering to the Principle of Controlling Schistosmiasis Japonica in Persistent Fashion

Schistosomiasis control is a systematic project with involvement of many parties, and characterized by long-term endeavors, hard work and complexity. Disease transmission will rebound if control efforts are relaxed. Therefore, China pursues long-term strategies for schistosomiasis japonica by intensifying control responsibilities of related public sectors, ensuring technical and financial support for control tasks, and assigning responsibility for control to leading roles of the government at each level, encouraging coordination and mutual support among administrative sections, and involving of relevant social parties. These measures, adopted after reaching the goal of transmission control in endemic provinces, can ensure final elimination of schistosomiasis japonica in China (MOH, 2008).

## Remaing Tasks and Future Directions in the Control of Schistosomiasis Japonica in Domestic Animals in China

To date, China has made remarkable achievements in schistosomiasis japonica control through over half a century of efforts. The prevalence of schistosomiasis japonica has fallen sharply in both humans and domestic animals. By 2015, the country reached the goal of transmission control, and is now moving toward the new control target: full elimination of schistosomiasis japonica in 2030, the promise specified in “Health China 2030” blueprint. In order to achieve the dream of “saying goodbye to the plague” and ensure safe living environments for the population, China will give priority to the following tasks in control of schistosomiasis japonica in domestic animals.

### Strengthening Transmission Risk Surveillance and Early Warning

Although China has reached the target to control transmission of schistosomiasis japonica, potential risks associated with epidemic of the disease have not been uprooted, because control of the infection sources was not completely implemented in some endemic areas; pasturing occasionally occurred in environments where *O. hupensis* is present; bovines were again raised in some areas following replacement with farm machines. These risks certainly will counteract the previous efforts and achievements, leading to unstable control of schistosomiasis in domestic animals, and present the chance of rebound or re-emergence of schistosomiasis japonica (Zhou et al., 2011; Lei and Zhou, 2014). In order to prevent such occurrences, China should make full use of the existing monitoring and warning networks, and put emphasis on surveillance in areas with underlying risks so that the potential rebound or re-emergence of schistosomiasis japonica is prevented and the previous achievements are preserved.

### Developing new Techniques for Targeted and Precise Control of the Disease

Currently, schistosomiasis japonica is present at a low endemic levels in China. This indicates that the original technologies for diagnosis, monitoring, and early warning systems may not be able to address the present needs of the disease prevention and control, not to speak of the requirements to achieve the goal for full elimination of the disease throughout the nation. Consequently, development of novel technology with higher diagnostic sensitivity and effectiveness in early warning are urgently needed. These measures should be dynamically informative of transmission species, addressing issues such as the quantity, distribution, and epidemic capacity of domestic animals. This may ensure timely release of the information for targeted control measures, toward consolidation of the previous efforts and arrival at the final elimination of schistosomiasis japonica (Xu et al., 2016; Zhou, 2016).

### Promoting Global Cooperation in Health and Sharing China’s Control Experiences

At present, schistosomiasis japonica still remains serious in the Philippines and Indonesia, and baseline data are not available in some areas of the two countries. The Philippines and Indonesia, each with a population of over 100 million, are both members of the Association of Southeast Asian Nations and important connections of China’s 21st-Century Maritime Silk Road. This implies that population migration will be more intensive between China and the two countries with increase of the economic trade, which will lead to new challenges in controlling infection through increased risk of imported cases. In the future, China has to intensify collaboration with the two countries in the field of schistosomiasis japonica control through sharing the successful experiences for the disease control so that the grand goal for full elimination of schistosomiasis japonica will be achieved in global scope.

## Perspectives

Schistosomiasis japonica is a zoonosis and its transmission is influenced by the environment. To effectively control and eliminate the disease in domestic animals, the traditional control pattern, namely relying on simple medication, veterinary medicine or modifying the environment to fight against the disease, is not appropriate because of the complexity of the transmission. More emphasis should be placed on interrupting transmission chains among humans, animals, and environmental space through multidisciplinary and integrated approaches to schistosomias japonica elimination by efforts from different sectors, including academic and research institutions, public or private sectors as well as non-governmental organizations and international agencies. In the control processes over 60 years, China persists in the tenet of One Health, into which human well-being, animal welfare and environment safety are incorporated, and in synchronous control of schistosomiasis japonica in humans and domestic animals, while weighing the importance of environment in the disease transmission. These measures and efforts are decisive roles in effective control of schistosomiasis japonica in China.

## Author Contributions

Drafted the article: ZC, revision of the article: ZC and YH, and final approval of the version to be published: ZC and TW.

## Conflict of Interest Statement

The authors declare that the research was conducted in the absence of any commercial or financial relationships that could be construed as a potential conflict of interest.
